# Correction: Structural frustration, metastability and cascade of polar phases in a layered lead bromide perovskite

**DOI:** 10.1039/d6sc90149b

**Published:** 2026-07-21

**Authors:** Mirosław Mączka, Katarzyna Fedoruk-Piskorska, Anna Gągor, Jan K. Zaręba, Dagmara Stefańska, Maciej Ptak, Dawid Drozdowski, Adam Sieradzki

**Affiliations:** a Institute of Low Temperature and Structure Research, Polish Academy of Sciences 50-422 Wrocław Poland m.maczka@intibs.pl; b Institute of Physics, University of Silesia Uniwersytecka 4 40-007 Katowice Poland; c Department of Experimental Physics, Wrocław University of Science and Technology Wrocław Poland; d Institute of Advanced Materials, Faculty of Chemistry, Wrocław University of Science and Technology 50-370 Wrocław Poland

## Abstract

Correction for ‘Structural frustration, metastability and cascade of polar phases in a layered lead bromide perovskite’ by Mirosław Mączka *et al.*, *Chem. Sci.*, 2026, https://doi.org/10.1039/d5sc09693f.

The authors regret that the affiliation listed for Adam Sieradzki was incorrect in the original article. The correct list of affiliations for this article is as shown herein.

The Royal Society of Chemistry apologises for these errors and any consequent inconvenience to authors and readers.

